# Effect of intense physical exercise on hepcidin levels and selected parameters of iron metabolism in rowing athletes

**DOI:** 10.1007/s00421-014-3018-3

**Published:** 2014-10-14

**Authors:** Anna Skarpańska-Stejnborn, Piotr Basta, Jerzy Trzeciak, Łucja Szcześniak-Pilaczyńska

**Affiliations:** 1 Department of Morphological and Health Sciences, Faculty of Physical Culture in Gorzów Wlkp., Gorzów Wielkopolski, Poland; 2 Department of Water Sports; Branch in Gorzów Wlkp., Faculty of Physical Culture, Gorzów Wielkopolski, Poland; 3 Department of Information and Tourism; Branch in Gorzów Wlkp., Faculty of Physical Culture, Gorzów Wielkopolski, Poland; 4 Department of Hygiene, University School of Physical Education, Poznan, Poland

**Keywords:** Strenuous exercise, Inflammation, Hemolysis, Iron

## Abstract

**Purpose:**

Physical exercise, especially intense physical exercise, causes a number of unfavorable changes, including an increase in the level of pro-inflammatory cytokines with the resultant sequestration of iron in macrophages and decreased iron absorption. This can lead to a reduced supply of iron for erythroid progenitor cells and promote the development of anemia.

**Method:**

This study included a group of 20 rowing athletes, members of the National Polish Rowing Team. The participants performed a 2,000-m maximum test on a rowing ergometer. Blood samples were taken from the antecubital vein prior to the exercise test, 1 min after completing the test, and after a 24-h recovery period. We determined the levels of hepcidin, interleukin 6 (IL-6), tumor necrosis factor* α*, soluble transferrin receptor, ferritin, total iron-binding capacity, unbound iron-binding capacity, iron, red blood cells, hemoglobin, hematocrit, mean corpuscular volume, creatine kinase, and myoglobin.

**Result:**

The high-intensity exercise test caused significant changes in hepcidin levels, IL-6, and iron metabolism parameters, with their subsequent return to baseline values during the recovery period. The serum iron levels decreased significantly during the recovery compared with pre- and post-exercise levels.

**Conclusion:**

These results suggest that the high-intensity ergometric test was reflected by a marked decrease in serum level of iron during the recovery period, but did not induce concomitant changes in the remaining erythrocyte parameters.

## Introduction

Physical exercise, especially in competitive sports, is characterized by considerable strain for an athlete, and causes faster ageing of the erythrocytes. Elevated body temperature, metabolic acidosis, hypoglycemia, and hemoconcentration, observed during physical exercise, reduce the osmotic resistance of erythrocytes (Reeder and Wilson 2001; Robinson et al. 2006). Yusof et al. (2007) suggested that intravascular hemolysis observed during long-term exercise results from the injury of older erythrocytes, which are less elastic and thus more susceptible to damage. The same authors documented a very strong inverse correlation (*r* = −0.911, *p* < 0.05) between hemolysis and the levels of spectrin, a peripheral protein of the erythrocyte membrane that is expressed on its internal surface and forms the cytoskeleton. These findings support the hypothesis that structural alterations of erythrocyte membranes increase the susceptibility of these cells to hemolysis, which results in elevated plasma levels of free iron.

Iron released from the cells can be stored in ferritin molecules, or bound to low-molecular-weight cytoplasmic ligands, forming the so-called labile iron pool (LIP). The iron contained in the LIP is further utilized to reconstruct iron-dependent enzymes or is involved in the generation of reactive oxygen species (Reeder and Wilson 2005). An increase in iron levels is associated with enhanced synthesis of hepcidin, a peptide that inhibits the secretion of iron from enterocytes and its release to plasma from monocytes and macrophages. Moreover, intensified binding of iron to its storage protein, ferritin, is observed in enterocytes (Deicher and Hörl 2006). Recent studies have revealed that hepcidin plays a pivotal role in the regulation of iron homeostasis, reducing its transport and absorption. This process is likely driven by the presence of enhanced hemolysis and acute phase response. The latter is associated with increased levels of pro-inflammatory cytokines, which in turn stimulate the synthesis of hepcidin (Van Deuren et al. 2009). Anemia of inflammation is characterized by normocytosis and normochromia, shorter survival of erythrocytes, low serum concentrations of iron and transferrin, and elevated levels of ferritin (Nikolaidis et al. 2003). Furthermore, increased iron content can be observed in macrophages of the reticuloendothelial system. The persistence of such status results in microcytosis and hypochromia. Notably, low levels of iron are a cause of reduced exercise capacity in athletes. It is estimated that a 1–2 g % decrease in blood hemoglobin concentration can lead to a 20 % reduction of exercise capacity (Gardner et al. 1977). Consequently, detailed analysis of exercise-induced changes in iron metabolism becomes vitally important.

Available data about the effect of short-term high-intensity exercise on hepcidin levels are sparse, although this type of load is a frequent component of sports training. Therefore, the aim of this study was to analyze the relationship between high-intensity physical exercise and changes in hepcidin levels and selected parameters of iron metabolism in rowing athletes.

## Materials and methods

### Study population

The study included 20 male athletes, members of the National Polish Rowing Team (16 heavyweight and 4 lightweight rowers), who took part in a training camp. All of the participants were free of any health problems and were not taking any anti-inflammatory drugs, vitamins, or medications known to affect iron metabolism for 2 weeks prior to the tests. The characteristics of the study participants are presented in Table 1. The Ethics Committee at the Poznań University of Medical Sciences approved the study protocol. All participants provided written informed consent. The participants were under no dietary restrictions.

**Table 1:** Basic characteristics of the study participants (means ± standard deviations)

Parameters	*N* = 20
Age, years	21.3 ± 0.82
Body mass, kg	87.7 ± 9.63
Body height, m	1.91 ± 0.06
Duration of training, years	7.2 ± 1.3

### Experimental procedure

The athletes performed a controlled 2,000 m time test. Each participant had to cover the distance on a rowing ergometer (Concept II, USA) in the shortest time possible. As the results of this test were taken into consideration during selection to the championship team, the athletes were well motivated to perform it to the best of their abilities. The test on a rowing ergometer was conducted in the morning hours, between 11:30 am and 12:00 pm. The air temperature during testing was 17 °C and relative humidity amounted to 55 %. Before the main test, each participant had 5 min for individual warm-up.

### Sample treatment

Blood samples from the athletes were obtained three times: (1) at rest (in the morning, after an overnight fast), (2) immediately after the exercise (1 min after completing the test), and (3) after a 24-h recovery period. Blood samples were obtained from the antecubital vein, with dipotassium ethylene diamine tetra-acetic acid (K_2_EDTA) used as an anticoagulant. Blood samples were taken for the analysis of red blood cells (RBCs), mean corpuscular volume (MCV), hemoglobin, and hematocrit in a MYTHIC 18 Hematology Analyzer (Orphee Medical, Geneva, Switzerland). Samples were immediately centrifuged to separate red blood cells from plasma and determine the activity of creatine kinase (CK).

Blood samples were collected without additives for the analysis of serum iron (Fe), soluble transferrin receptor (sTfR), ferritin, total iron-binding capacity (TIBC), interleukin (IL)-6, tumor necrosis factor (TNF)-α, hepcidin, and myoglobin. Serum and plasma samples were frozen immediately after centrifugation and stored at −80 °C until use. Additionally, capillary blood samples were obtained from an earlobe prior to warm-up preceding the ergometric test and after (within 1 min) each exercise test to assess the athletes’ lactic acid levels.

### Measurements

Serum IL-6 was measured using a commercially available enzyme-linked immunosorbent assay (ELISA; Quantikine HS, R&D Systems, Minneapolis, Minnesota, USA) with an assay range of 0.38–10 pg ml^−1^. The average interassay CV precision was 5.4 %. Serum concentrations of TNF-α (in pg/ml) were quantified using a commercially available enzyme immunoassay (Quantikine human immunoassay, cat. no. DTA00C, R&D Systems Inc., Minneapolis, Minnesota, USA). The average interassay CV precision was 7.6 %. Serum hepcidin was measured using a commercially available ELISA (Wuhan EIAab Science Co., China) with an assay range of 0.187–12 ng ml^−1^. The average interassay CV precision was 2.7 %.

Iron concentration and TIBC were measured using the colorimetric method with chromogens (cat. no. SI257 and TI1010, Randox, Antrim, UK); the results were expressed in µg/dL. The unsaturated iron-binding capacity (UIBC) was calculated from the formula: UIBC = TIBC–Fe. The myoglobin concentration was determined immunochemically, with the help of the Myoglobin ELISA kit (cat. no. 11170, OxisResearch, Percipio Bio, Foster City, California, USA); the results were expressed in ng/mL. Serum ferritin levels were determined immunochemically, with an aid of a commercially available diagnostic kit (cat. no. DE7750, Hölzel Diagnostika, Köln, Germany); the results were expressed in ng/mL. The average interassay CV precision was 7.5 %. Concentrations of soluble transferrin receptor (sTfR) were determined immunochemically with a commercially available diagnostic kit (cat. no. RD194011100, BioVendor, Brno, Czech Republic). The average interassay CV precision was 4.3 %. Creatine kinase (CK) activity was determined in plasma samples with a commercially available kit (cat. no. LCN 282, HACH LANGE, Düsseldorf, Germany); the results were expressed in U/L. The lactic acid in capillary blood was determined immediately after sample collection using a commercially available kit (cat. no. LKM 140, HACH LANGE, Düsseldorf, Germany); lactic acid concentrations were expressed in mmol/L. If necessary, the results were adjusted for hemoconcentration, using the exercise-induced changes in hematocrit as a covariate.

### Statistical analysis

Statistical analyses were performed using the STATISTICA v. 10.0 software package (Stat–Soft, Cracow, Poland). Data distribution was analyzed using the Shapiro–Wilk test. Homogeneity of variance was verified with Levene’s test. If significant changes were documented in one-way analysis of variance (ANOVA), Fisher’s post hoc test was applied to locate the source of significant differences. The non-parametric Friedman ANOVA was used in the case of variables with distributions other than normal. Pearson’s coefficients of linear correlation were calculated for correlation analysis. All values were reported as mean ± SD. The statistical significance of all the tests was set at *p* ≤ 0.05.

## Results

Physical characteristics and duration of training are shown in Table 1. Power output, blood lactate levels, and total run time are shown in Table 2. Data on a training load of the examined athletes during 30 days preceding the ergometric test are presented in Table 3.

**Table 2:** Power output, blood lactic acid levels, and total run time (means ± standard deviations)

Parameters	*N* = 20
Power, watts	430 ± 34.1
W/kg	4.88 ± 0.28
LA_min_, mmol/L	1.6 ± 0.14
LA_max_, mmol/L	14.9 ± 2.62
Time, s	373.4 ± 10.38

**Table 3:** Training schedule during the 3 days preceding blood sample collection

	Days before ergometer test			
	3	2	1	
Total training time, min/day	210	150	120	Ergometer test—2,000 m
Time rowed, min/day	70	90	100	
Distance rowed, km/day	16	18	20	
Training for force development, min/day	70			
Extensive endurance rowing training time, min/day	40	90	100	
High-intensity endurance rowing training time, min/day	30			
Unspecific training (running, etc.), min/day	70	60	20	

**Fig. 1:**
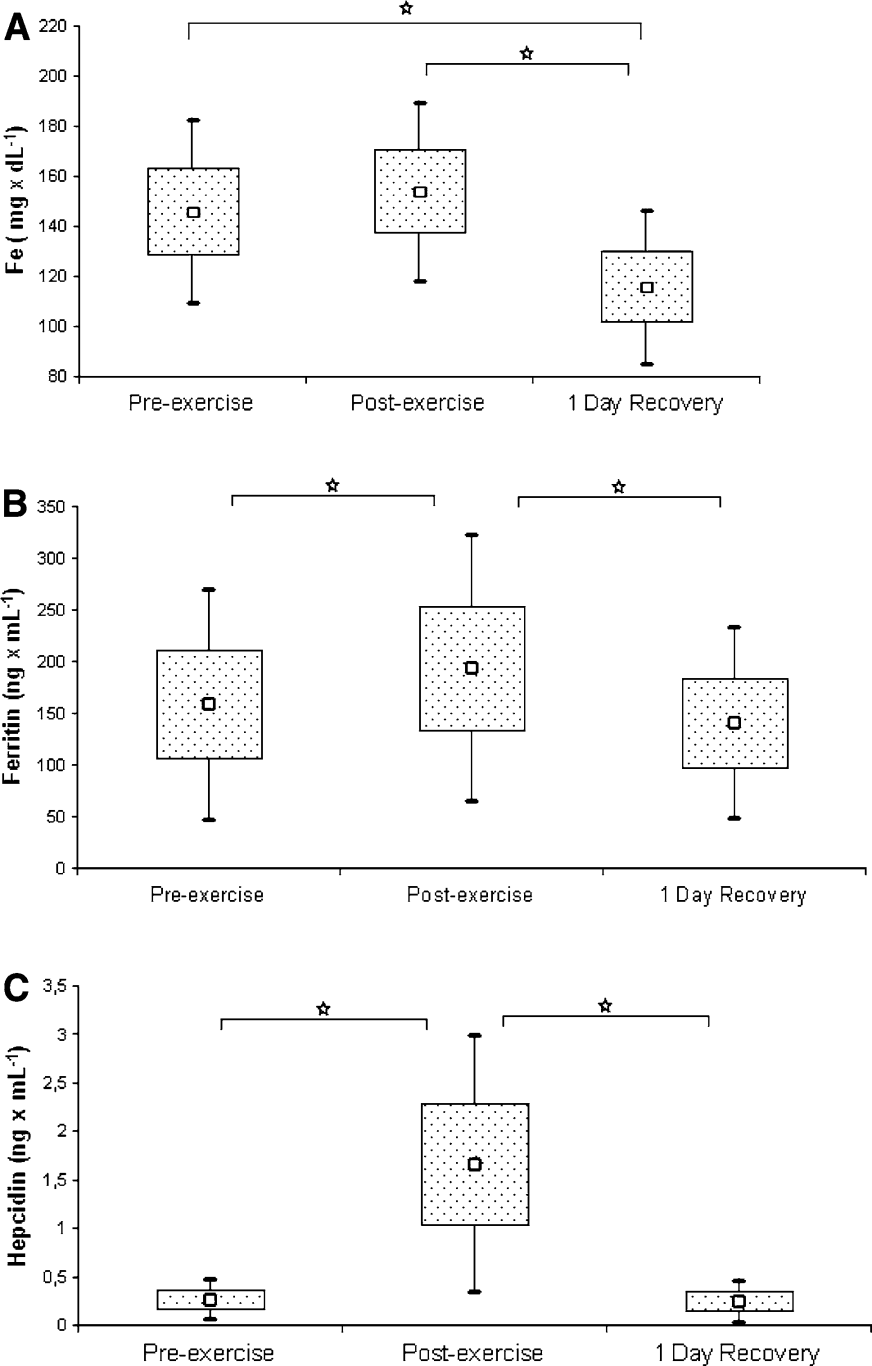
Serum iron **a**, ferritin **b**, and hepcidin **c** levels in rowers at baseline, immediately after exercise, and after a 1-day recovery period. Data are presented as mean ± SEM. *Statistically significant difference between trials (*p* < 0.05)

The values of blood parameters that indicate iron metabolism in the studied athletes are presented in Table 4 and Fig. 1 (a: iron, b: ferritin, and c: hepcidin). Participation in the high-intensity exercise test resulted in a significant increase in most of the studied parameters, and their subsequent return to pre-exercise levels after 1 day of recovery. However, an exercise-induced increase in serum iron level proved insignificant during statistical analysis, and the values for this parameter (documented at the end of the recovery period) were significantly lower than its baseline and post-exercise measurements.

**Table 4:** Changes in iron metabolism status during exhaustive exercise

Variables	Pre-exercise M (± SD)	Post-exercise M (± SD)	Recovery M (± SD)	*p* (ANOVA)
RBC, 10^12^/L	5.29 (0.29)	5.65 (0.30)^a^	5.26 (0.30)^c^	0.00000
HGB, g/dL	15.47 (0.67)	16.54 (0.81)^a^	15.34 (0.67)^c^	0.00000
HCT, %	44.33 (1.95)	48.23 (2.09)^a^	43.99 (2.26)^c^	0.00000
MCV, fL	85.78 (2.69)	87.30 (2.87)^a^	85.51 (2.79)^c^	0.00000
TIBC, µg/dL	320.8 (32.83)	352.2 (34.57)^a^	325.5 (26.82)^c^	0.00155
UIBC, µg/dL	175.0 (17.92)	192.1 (18.87)^a^	177.6 (14.66)^c^	0.00155
sTfR, µg/mL	0.93 (0.14)	1.26 (0.24)^a^	0.93 (0.17)^c^	0.00002
Myoglobin, ng/mL	245.2 (71.38)	407.7 (131.21)^a^	373.7 (104.86)^b^	0.00026

*RBC* red blood cells, *HGB* hemoglobin, *HCT* hematocrit, *MCV* mean corpuscular volume, *UIBC* unsaturated iron-binding capacity, *TIBC* total iron-binding capacity, *sTfR* soluble transferrin receptor

^a^Pre-exercise–post-exercise

^b^Pre-exercise–recovery

^c^Post-exercise–recovery

**Fig. 2:**
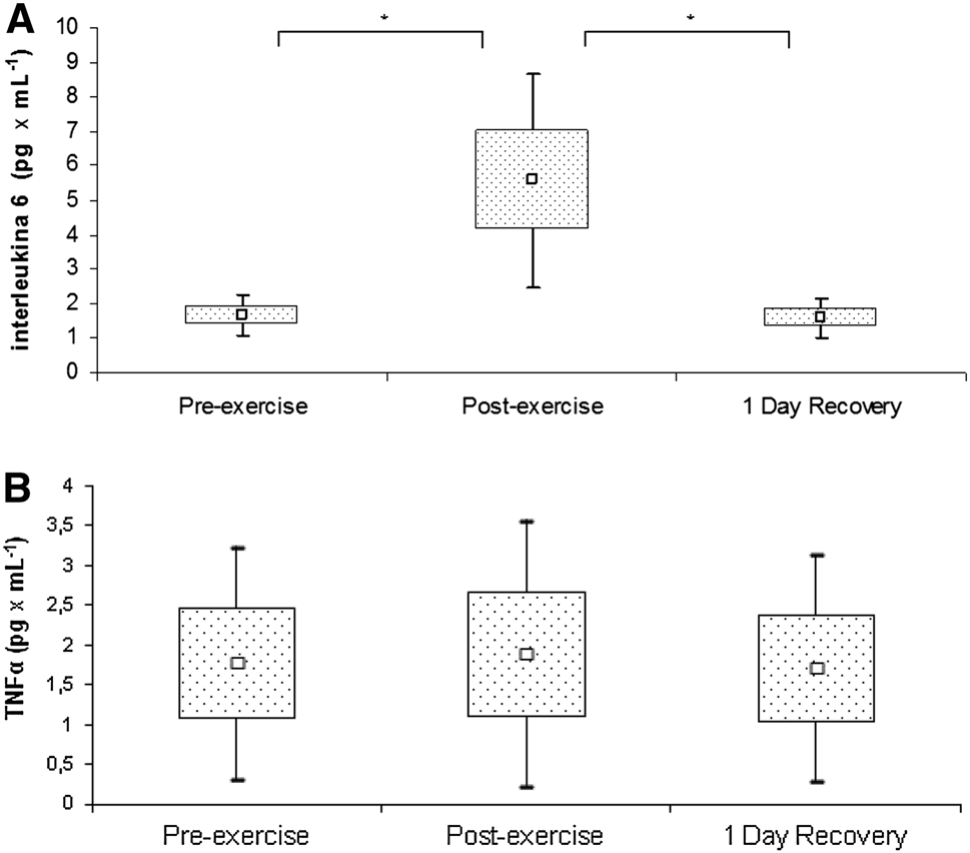
Interleukin 6 **a** and tumor necrosis factor α **b** levels in rowers at baseline, immediately after exercise, and after a 1-day recovery period. Data are presented as mean ± SEM. *Statistically significant difference between trials (*p* < 0.05)

Furthermore, the exercise test resulted in an increase in serum IL-6. Upon recovery, this parameter returned to its pre-exercise levels (Fig. 2a). In contrast, we did not document a significant effect of physical exercise on TNF-α levels (Fig. 2b).

**Fig. 3:**
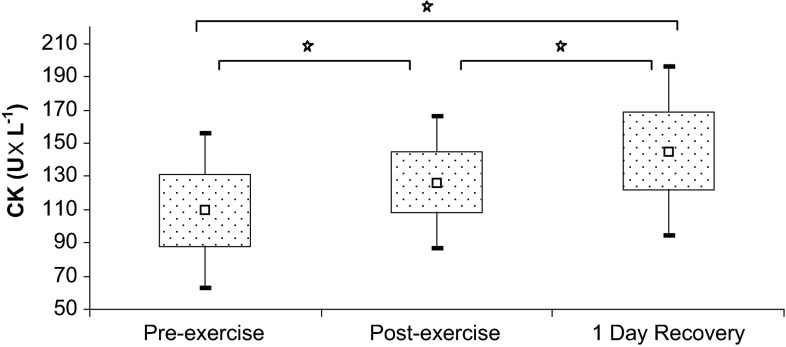
Creatine kinase levels at baseline, immediately after exercise, and after a 1-day recovery period. Data are presented as mean ± SEM. *Statistically significant difference between trials (*p* < 0.05)

Finally, creatine kinase activity (Fig. 3) and serum myoglobin levels (Table 4) increased significantly in response to the exercise test and remained elevated after 1 day of recovery.

## Discussion

Enhanced inflammatory response, observed during intense exercise load, causes the secretion of many mediators from circulating macrophages, including IL-6, which stimulates the expression of hepcidin in hepatocytes and its release into the circulation (Banzet et al. 2012). This phenomenon was confirmed in our study. The ergometric exercise test caused a significant increase in IL-6 and hepcidin levels, along with a 22 % increase in ferritin, a marker of systemic iron deposits that is classified as an acute phase protein. We also observed a positive correlation between IL-6 and hepcidin levels (*r* = 0.481, *p* < 0.05). Antosiewicz et al. (2013) studied trained and untrained individuals exposed to the high-intensity interval exercise test, and observed post-exercise increases in IL-6 and hepcidin in both groups. However, the hepcidin levels in trained participants returned to baseline, pre-exercise values as soon as after 1 day of recovery, versus after as many as 5 days of recovery for untrained individuals. These results are consistent with the findings documented for our rowers, in whom the concentrations of IL-6 (Fig. 3a) and hepcidin (Fig. 1c) returned to baseline values 1 day post-exercise.

In contrast to our findings regarding IL-6, we did not document any significant changes in TNF-α concentration, either in response to exercise stimulation or during the recovery period (Fig. 3b). Rämson et al. (2008) showed that post-exercise TNF-α concentrations in rowers are also significantly modulated by the training load before the trial. The post-exercise increase in this cytokine was observed solely during the period of high-volume training. It is noteworthy that we examined our rowers during the general preparation phase, which is predominated by aerobic exercise (Table 3) and probably insufficient to induce changes in TNF-α concentration.

According to available data, the mechanism that controls the post-exercise expression of hepcidin is associated not only with enhancement of the inflammatory response, but also with a hemolysis-induced increase in iron levels (Reeder and Wilson 2005). While the ergometric test resulted in only an insignificant increase in serum iron concentrations among our rowers (Fig. 1a), the remaining parameters that characterize systemic iron metabolism (TIBC, UIBC, sTfR) increased significantly.

According to Barros et al. (2012), ferritin (and probably also transferrin), but not hemolysis or rhabdomyolysis, can constitute the main sources of free iron during physical exercise. Higher post-trial levels of CK and myoglobin observed in our rowers confirmed the presence of exercise-induced injury to the sarcolemma. However, a further increase in these parameters during the recovery period (Fig. 3; Table 4) did not result in corresponding changes of the remaining parameters of iron metabolism; this finding is consistent with the above-mentioned hypothesis regarding the regulatory roles of hepcidin and ferritin. According to Suedekum and Dimeff (2005), an increase in ferritin concentration can result from the enhanced permeability of cellular membranes associated with exercise-induced injury of the reticuloendothelial system cells and hepatocytes. The latter cells exhibit enhanced ferritin synthesis in response to erythrocyte hypercatabolism or mild hemolysis. Despite being stable in acute conditions (Das Gupta and Abbi 2003), the second marker of iron metabolism, a soluble transferrin receptor, is less useful as a measure of exercise-induced changes in iron status owing to its lack of standardization. We observed a 26 % post-exercise increase in sTfR concentration among our rowers, as well as its subsequent return to baseline during the recovery period (Table 4).

A 24-h recovery period resulted in a marked decrease in iron level in our participants, compared with both pre- and post-exercise levels (Fig. 1a). Reinke et al. (2012) observed that a considerable proportion of elite rowing athletes and professional soccer players have iron deficiency, independent of the training mode. However, only rowers exhibit lower erythrocyte counts and hemoglobin levels during pre-season training than during the recuperation period. According to Nemeth et al. (2004), impaired hemoglobin synthesis can result from a transient increase in hepcidin activity, which causes rapidly decreasing plasma iron levels and resultant anemia.

Liu et al. (2006) showed that high-intensity exercise causes a decrease in the serum level of iron in rats; in contrast, serum iron was significantly increased in animals exposed to moderate exercise. Decreased iron ion concentration in biological fluids, including blood serum, can constitute one of basic protective mechanisms that is activated in order to prevent their involvement in reactions associated with the generation of toxic oxygen species.

A study of iron-injected mice exposed to a treadmill test demonstrated a number of unfavorable changes that were not observed in control animals. The iron-overloaded mice exhibited significantly higher iron content in muscles and plasma, which (according the authors) was caused by enhanced oxidative stress. Furthermore, the excessive, rapid accumulation of iron exerted negative effects on skeletal muscle function in mice, as confirmed by decreased muscle strength and the presence of muscle atrophy (Reardon and Allen 2009). Similarly, 1 week of excessive iron supplementation (120 mg/day) resulted in increased oxidative stress and inflammatory response in young, healthy humans (Schümann et al. 2005).

Intense physical exercise on a rowing ergometer resulted in increases in all analyzed erythrocyte parameters of our rowers, with their subsequent return to baseline after a 1-day recovery period (Table 4). Boyadjiev and Taralov (2000) showed that highly trained athletes from different sports have lower red blood cell counts, packed cell volumes, and hemoglobin concentrations than untrained controls. Moreover, they observed that the values of these parameters were the lowest among male swimmers and rowers (i.e., representatives of disciplines characterized by high-intensity training), making them predisposed to so-called sports anemia. It is noteworthy that depending on the training phase, youth rowers perform between 15 and 25 h of exercise per week. Maintaining proper balance between intensive exercise and recovery appears to be vitally important in view of such a considerable training load. Too heavy an exercise load and too short a recovery period can lead to the accumulation of unfavorable training outcomes, which manifests as, among other things, a decrease in erythrocyte parameters (Rietjens et al. 2005).

Analyzing the results of previous studies, one can hardly identify the hematological parameters that might be helpful in diagnosing iron deficiency in athletes. Chronic exercise-induced inflammation is associated with iron deficiency, which should be distinguished from true anemia. Typically, determined hematological parameters (erythrocyte count, packed cell volume, serum iron concentration) do not fully reflect the degree and severity of sports anemia. In turn, the levels of ferritin and transferrin are not sufficiently accurate, as they are both elevated in any anemia of inflammation. Finally, sTfR concentration, which is elevated in acute states, constitutes a marker of iron deficiency in tissues, rather than a measure of anemia (Skikne 2008; Zoller and Vogel 2004). We observed a 26 % post-exercise increase in sTfR concentration in our rowers, with its subsequent return to baseline during the recovery period (Table 4). According to Schumacher et al. (2002), the post-test increase in sTfR is determined by exercise intensity. In their study, both trained and untrained individuals exhibited increased sTfR solely in response to the more intense exercise test.

## Conclusions

A high-intensity exercise test caused significant changes in the hepcidin levels and parameters of iron metabolism of rowing athletes, which subsequently returned to baseline values during the recovery period. Serum iron levels decreased significantly during the recovery period. While a single bout of intense exercise did not cause clinically significant loss of iron in our athletes, the consequences of two to three daily overexertion loads (e.g. competition or period of intense load) can accumulate and constitute an important risk factor for anemia. Future studies should be aimed at determining the time that is required to return iron levels to their pre-exercise values and identifying factors that promote the replenishment of iron resources in athletes.

## Abbreviations

ANOVA: Analysis of variance
CK: Creatine kinase
Fe: Serum iron
HCT: Hematocrit
HGB: Hemoglobin
IL-6: Interleukin
K_2_EDTA: Dipotassium ethylene diamine tetra-acetic acid
LA: Lactic acid
LIP: Labile iron pool
MCV: Mean corpuscular volume
RBCs: Red blood cells
STfR: Soluble transferrin receptor
TIBC: Total iron-binding capacity
TNF-α: Tumor necrosis factor alpha
UIBC: Unsaturated iron-binding capacity
VO_2peak_: Peak oxygen consumption
